# Sugarcane Water Stress Tolerance Mechanisms and Its Implications on Developing Biotechnology Solutions

**DOI:** 10.3389/fpls.2017.01077

**Published:** 2017-06-23

**Authors:** Thais H. S. Ferreira, Max S. Tsunada, Denis Bassi, Pedro Araújo, Lucia Mattiello, Giovanna V. Guidelli, Germanna L. Righetto, Vanessa R. Gonçalves, Prakash Lakshmanan, Marcelo Menossi

**Affiliations:** ^1^Functional Genome Laboratory, Department of Genetics, Evolution and Bioagents, Institute of Biology, State University of CampinasCampinas, Brazil; ^2^Sugar Research AustraliaBrisbane, QLD, Australia

**Keywords:** drought, sugarcane, transgenic, bioethanol, abscisic acid, lipid peroxidation

## Abstract

Sugarcane is a unique crop with the ability to accumulate high levels of sugar and is a commercially viable source of biomass for bioelectricity and second-generation bioethanol. Water deficit is the single largest abiotic stress affecting sugarcane productivity and the development of water use efficient and drought tolerant cultivars is an imperative for all major sugarcane producing countries. This review summarizes the physiological and molecular studies on water deficit stress in sugarcane, with the aim to help formulate more effective research strategies for advancing our knowledge on genes and mechanisms underpinning plant response to water stress. We also overview transgenic studies in sugarcane, with an emphasis on the potential strategies to develop superior sugarcane varieties that improve crop productivity in drought-prone environments.

## Introduction

Environmental stresses limit plant growth and crop productivity (Mahajan and Tuteja, 2005; Lobell et al., 2011). Drought is considered to be the most deleterious abiotic stress, affecting crop productivity worldwide (Wang et al., 2003; Rampino et al., 2006). Sugarcane, an important source of sugar and ethanol, is a relatively high water-demanding crop and its growth is highly sensitive to water deficit (Lakshmanan and Robinson, 2014). It is estimated that sugarcane produces 8–12 ton cane per ML of irrigation water (Kingston, 1994), and water deficit can lead to productivity losses up to 60% (Robertson et al., 1999; Ramesh, 2000; Basnayake et al., 2012; Gentile et al., 2015). For this reason, production areas are concentrated in regions with favorable rain regime to sugarcane growth and development (Moreira et al., 2007), while in other areas crop production requires supplemental or full irrigation (Walter et al., 2013).

The increasing incidence, duration and intensity of severe water deficit, has prompted many large sugarcane crop improvement programs to invest in water use-efficient and water stress tolerant varieties and water use-efficient crop productions systems. The increasing knowledge of stress biology coming from genetic, agronomic and molecular biology studies in various crops, including sugarcane is providing a major impetus to develop biotechnological strategies for producing water stress tolerant and commercially useful sugarcane varieties (Lakshmanan and Robinson, 2014; Augustine et al., 2015c; Ramiro et al., 2016). Plants have evolved various drought tolerance strategies, such as changes in life cycle, modulation of growth and development to match with water supply, regulation of whole plant functions to balance resource allocation for growth and stress adaptation, and evolution of stress signal perception for rapid and long-term expression of stress tolerance (Hirayama and Shinozaki, 2010; Hu and Xiong, 2014; You and Chan, 2015). The expanding knowledge base helped to identify key genes associated with drought tolerance and maintenance of growth under water deficit condition in various crops including sugarcane (Wang et al., 2003, 2016; Yamaguchi and Blumwald, 2005; Hu and Xiong, 2014; Augustine et al., 2015c; Ramiro et al., 2016). Biotechnology and molecular breeding techniques are useful tools to enhance crop productivity under drought stress. Despite the availability of molecular tools and strategies and advancements in our understanding of stress responses, engineering crops for drought tolerance remains a major challenge (Wang et al., 2003, 2016; Hu and Xiong, 2014). This is not only due to the complexity of the plant responses to water deficit (Hu and Xiong, 2014; Wang et al., 2003, 2016), but also due to the difficulty of identifying and exploting large effect genes and alleles and the associated selection traits for developing drought tolerant varaties suitable for commercial crop production conditions (Tardieu, 2012; Cominelli et al., 2013).

The objective of this review is to report recent advances in our understanding of water stress-response mechanisms in sugarcane from molecular, biochemical and physiological perspectives, and highlight what we consider the most promising strategies for developing drought tolerant sugarcane.

## Morphological and physiological responses of sugarcane to water stress

Sugarcane development is broadly divided into three stages: germination, plant establishment and early tillering phase, grant vegetative growth phase, and maturation and flowering. From a water stress management perspective germination, tillering and grant vegetative growth stage were the target of many studies because they are important growth phases from a crop production perspective. The susceptibility of sugarcane to water stress is greater in the tillering and stem elongation phases (Inman-Bamber and Smith, 2005; Machado et al., 2009) with both stem and leaf growth are most affected than other organs (Ramesh, 2000; Machado et al., 2009; Lakshmanan and Robinson, 2014). However, moderate water stress at maturation phase has positive effects on sucrose yield, since photosynthesis is less sensitive to water stress than stem growth, channeling assimilated CO_2_ to sucrose production and accumulation in the stalk (Inman-Bamber, 2004).

**Figure 1:**
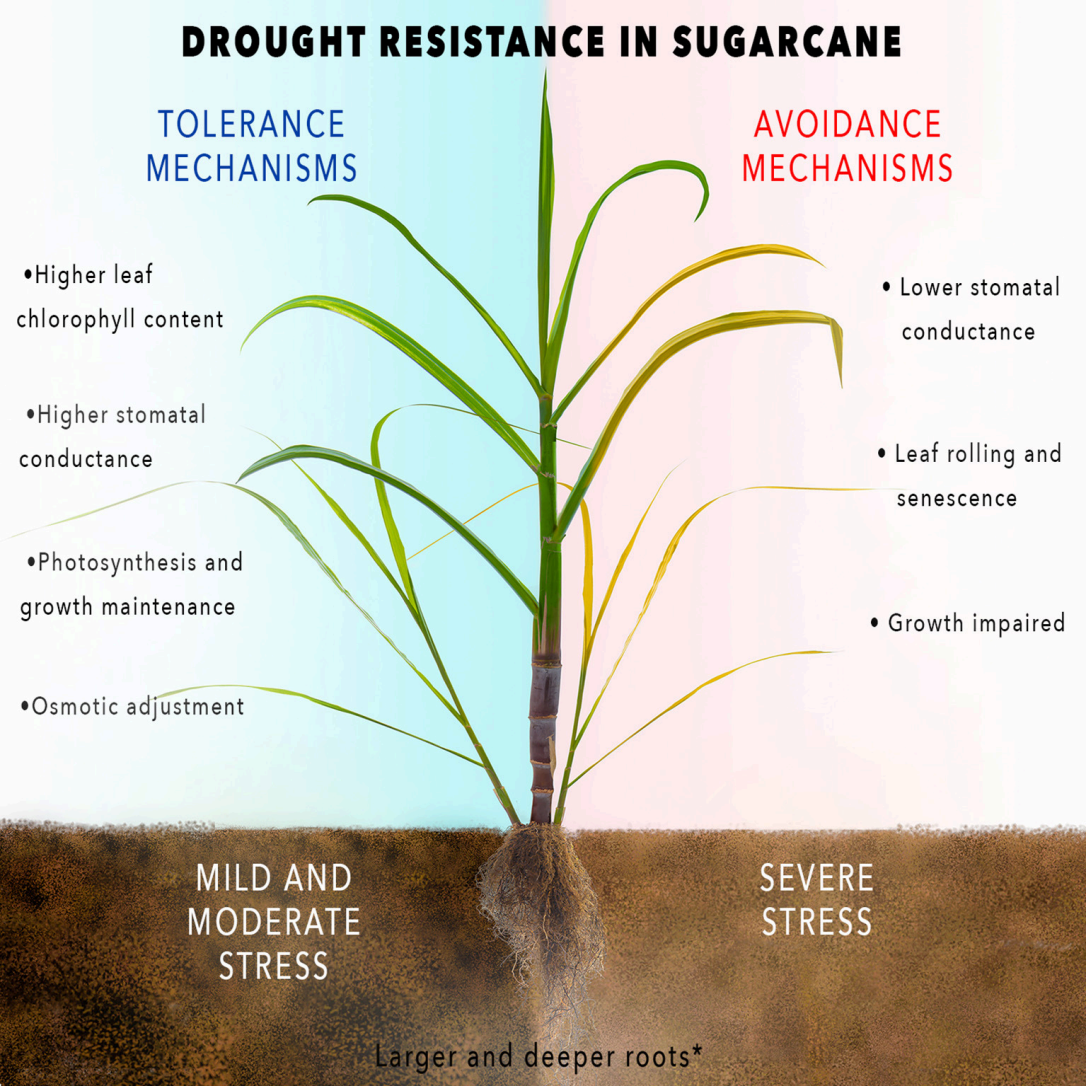
Scheme of sugarcane drought-response mechanisms.

Severe water stress, drought, affects the entire plant (Figure 1). Morphological and physiological responses of sugarcane plants vary according to the genotype, the duration (rapid or gradual) and the intensity (severe or mild) of stress and also the type of the tissue affected (Bartels and Sunkar, 2005; Smit and Singels, 2006; Da Graça et al., 2010; Inman-Bamber et al., 2012). As mentioned above, water stress also affects both cane and sugar yield substantially. However, large potentially exploitable genetic variation for cane and sugar yield under water stress has been reported (Hemaprabha et al., 2004, 2006; Basnayake et al., 2012). The most common water stress responses in sugarcane are leaf rolling, stomatal closure, inhibition of stalk and leaf growth, leaf senescence and reduced leaf area (Inman-Bamber and Smith, 2005; Inman-Bamber et al., 2012). Moreover, under water stress, both cell division and cell elongation are interrupted (Machado et al., 2009) and stem and leaf elongation are the most seriously affected growth processes (Inman-Bamber, 2004; Inman-Bamber et al., 2008). Root development is also influenced by water deficit (Inman-Bamber and Smith, 2005; Smit and Singels, 2006), but relatively less than the above-ground biomass.

Sugarcane is a tropical crop with C_4_ photosynthetic metabolism. Under moderate water stress a decrease in stomatal conductance (*g*_*s*_), transpiration rate (*E*), internal CO_2_ concentration (Ci), and photosynthetic rate occurs, mainly due to stomatal limitations (Du et al., 1996; Inman-Bamber and Smith, 2005; Silva et al., 2007; Da Graça et al., 2010; Endres et al., 2010; da Silva et al., 2012; Medeiros et al., 2013; Basnayake et al., 2015). This, along with stalk and leaf growth inhibition, are the most common initial adaptation when sugarcane plants are subjected to mild to moderate dehydration (Inman-Bamber and Smith, 2005). However, water stress-induced non-stomatal limitations have also been reported as cause of photosynthesis inhibition in sugarcane (Ribeiro et al., 2013). This often occurs when stress is severe or under prolonged moderate water deficit conditions (Basnayake et al., 2015). Water stress induced decline in photosynthetic rate is mainly caused by a decrease in phosphoenolpyruvate carboxylase (PEPcase) and ribulose-1,5-biphosphate carboxylase (Rubisco) activity (Du et al., 1996; Inman-Bamber and Smith, 2005; Lakshmanan and Robinson, 2014). It is worth to note that photosynthesis rate is also impacted by sugar accumulation in the leaves (McCormick et al., 2008). Under non-stressed condition low leaf sugar content is conducive to photosynthesis, while high sugar content moderates carbon fixation (Goldschmidt and Huber, 1992).

Interestingly, increased levels of some sugars, such as trehalose, can help plants to cope with water deficit, reducing the damage to cell membrane (Delorge et al., 2014). The capacity to accumulate trehalose was demonstrated in sugarcane roots under drought. Sales et al. (2012) reported an increase in starch hydrolysis, leading to higher levels of soluble sugars that helped sustain carbon supply even in a reduced CO_2_ fixation condition, facilitating growth recovery after stress.

## Water stress tolerant sugarcane

Understanding the nature of the agricultural problem from both genetic and crop production perspectives is critical for developing effective and commercially applicable solutions (Blum, 2005). Stress is defined as any restriction of normal functions and development that plants have to confront during their life cycles. In order to survive and grow under stress, plants have evolved different adaptive mechanisms, which comprise broadly the concepts of escape, avoidance and tolerance (Levitt, 1980):

- Escape mechanisms include escaping the adverse conditions by rapid development to complete life cycle before stress sets in Kooyers (2015). Because sugarcane is a perennial crop, this strategy does not apply for this species.
- Dehydration avoidance involves mechanisms to sustain high water status or cellular hydration through low stomatal conductance during stress condition (Blum, 2005; Kooyers, 2015).
- Dehydration tolerance is referred to as mechanisms that allow plants to tolerate stress and maintain plant functions under water deficit (Levitt, 1980; Blum, 2005).

These mechanisms are not mutually exclusive and the same plant can resort to combinations of such strategies (Figure 1; Pimentel, 2004; Bueno et al., 2006). Avoidance mechanisms are important traits in areas with severe or terminal water deficit because they enhance the chances of capturing maximum soil moisture, limiting water loss and retaining cellular hydration, thereby, allowing crop recovery when stress is relieved. However, under severe water stress these mechanisms reduce biomass accumulation through large reduction in transpiration, leaf area, and carbon fixation (Blum, 2005; Tardieu, 2012; Cominelli et al., 2013). On the other hand, tolerance mechanisms are favorable traits under mild and moderate water deficit conditions, because these mechanisms allow growth maintenance during the stress. Tolerance traits are directly linked to high stomatal conductance, sustaining the photosynthesis rate and also heat stress tolerance by decreasing leaf temperature (Blum, 2005; Tardieu, 2012; Cominelli et al., 2013).

The following section presents an overview of morphological and physiological alterations that have been considered as useful traits to differentiate susceptible and tolerant sugarcane genotypes by researchers and breeders (Tables 1, 2). It is important to note that in water stress studies tolerance and resistance terminologies are used synonymously, which is incorrect. In this review, we use the concept of tolerance to describe plant and crop response to water stress as there is no absolute resistance to water stress without adverse growth or other plant function exits in any plant or crop.

**Table 1:** Morphological traits used to differentiate the degrees of drought resistance in sugarcane genotypes.

**Morphological trait**	**Age (DAP)**	**Stress applied**	**Time of stress (Days)**	**Experimental conditions**	**References**
Number of tiller	60	Water withholding	90	F	Venkataramana et al., 1986; Silva et al., 2008
Stalk diameter and length	55; 60; 90	Water withholding; 7 cycles of 4 days (water supply at first day followed by 3 days of water withholding)	12; 28; 90	G/F	Wagih et al., 2003; Silva et al., 2008; Hemaprabha et al., 2013; Zhao et al., 2013
Single stalk weight	60	Water withholding	90	G / F	Silva et al., 2008; Hemaprabha et al., 2013
Internode length and weight	60	Water withholding	90	G/F	Silva et al., 2008; Hemaprabha et al., 2013
Number of internodes	60	Water withholding	90	G/F	Silva et al., 2008; Hemaprabha et al., 2013
Shoot dry mass	60; 83	Water withholding	4; 25	G	Medeiros et al., 2013; Ribeiro et al., 2013
Root dry mass	60; 100	Water withholding	4; 10	G	Jangpromma et al., 2012; Medeiros et al., 2013
Leaf parameters (length, width and number of green leaves)	90	7 cycles of 4 days (water supply at first day followed by 3 days of water withholding)	28	G	Wagih et al., 2003
Root parameters (length, surface area and volume)	100	Water withholding	10	G	Jangpromma et al., 2012

*DAP, days after planting; ET, evapotranspiration; G, greenhouse; F, field*.

**Table 2:** Physiological traits used to differentiate the degrees of drought resistance in sugarcane genotypes.

**Physiological trait**	**Age (DAP)**	**Stress applied**	**Time of stress (Days)**	**Experimental conditions**	**References**
Photosynthetic rate, stomatal conductance and transpiration rate	15; 55; 60; 83; 180	Water withholding; 80% of water lost by (ET); 20% of available water; 50% capacity for the pots water retention	4; 12; 15; 25; 60; 70	G/F	Du et al., 1996; Da Graça et al., 2010; Endres et al., 2010; da Silva et al., 2012; Medeiros et al., 2013; Ribeiro et al., 2013; Silva et al., 2013; Zhao et al., 2013
PSII (photosystem II quantum yield)	15; 60; 83; 180	Water withholding; 20% of available water	25; 70; 90	G/F	Silva et al., 2007; Da Graça et al., 2010; da Silva et al., 2012; Ribeiro et al., 2013; Silva et al., 2013
Activity of enzymes of photosynthetic apparatus	15	Water withholding	4	G	Du et al., 1996
Leaf pigments content (*chlorophylls and carotenoids)*	15; 60; 55; 180	Water withholding; 20% of available water; 50% of capacity for the pots water retention	4; 12; 70; 90	F/G	Du et al., 1996; Silva et al., 2007; da Silva et al., 2012; Medeiros et al., 2013; Silva et al., 2013; Zhao et al., 2013
Leaf temperature^*^	15; 180	20% of available water	70; 90	G/F	Silva et al., 2007; Da Graça et al., 2010; da Silva et al., 2012
Leaf water potential	15; 60; 83; 180	Water withholding; 20% of available water; 50% of capacity for the pots water retention	4; 25; 70; 90	G/F	Du et al., 1996; Silva et al., 2007; Da Graça et al., 2010; Endres et al., 2010; da Silva et al., 2012; Medeiros et al., 2013; Ribeiro et al., 2013; Silva et al., 2013

DAP, days after planting; ET, evapotranspiration; G, greenhouse; F, field;

* *Increase under drought*.

As discussed above, dehydration tolerance mechanisms help achieve better growth and crop yield. Although the exact mechanism(s) of water stress tolerance is not understood in sugarcane, some traits have been implicated to a better performance of crops under mild to moderate stress. For example, Silva et al. (2008) concluded that higher productivity under stress is associated with higher stalk number, stalk height and stalk weight. On the other hand, stalk diameter is variable among varieties, being more dependent on the genotype than the environment (Da Silva and Da Costa, 2004; Soares et al., 2004; Silva et al., 2008). Leaf chlorophyll content (SPAD index), leaf and canopy temperature, photosynthesis rate, stomatal conductance (*g*_*s*_), canopy conductance (*g*_*c*_), and transpiration rate (*E*) are also used as an indirect selection criteria for sugarcane genotypes tolerant to water stress (Silva et al., 2007; Endres et al., 2010; da Silva et al., 2012; Basnayake et al., 2015). Retention of green leaf area, also termed as “stay-green” phenotype, is also considered as an important characteristic for sustaining yield potential (Thomas and Howarth, 2000; Blum, 2005). In general, genotypes with higher stomatal and canopy conductance, lower leaf and canopy temperature, and consequently higher transpiration under mild to moderate water stress condition are regarded as tolerant with potential for deploying as commercial varieties or for breeding.

**Figure 2:**
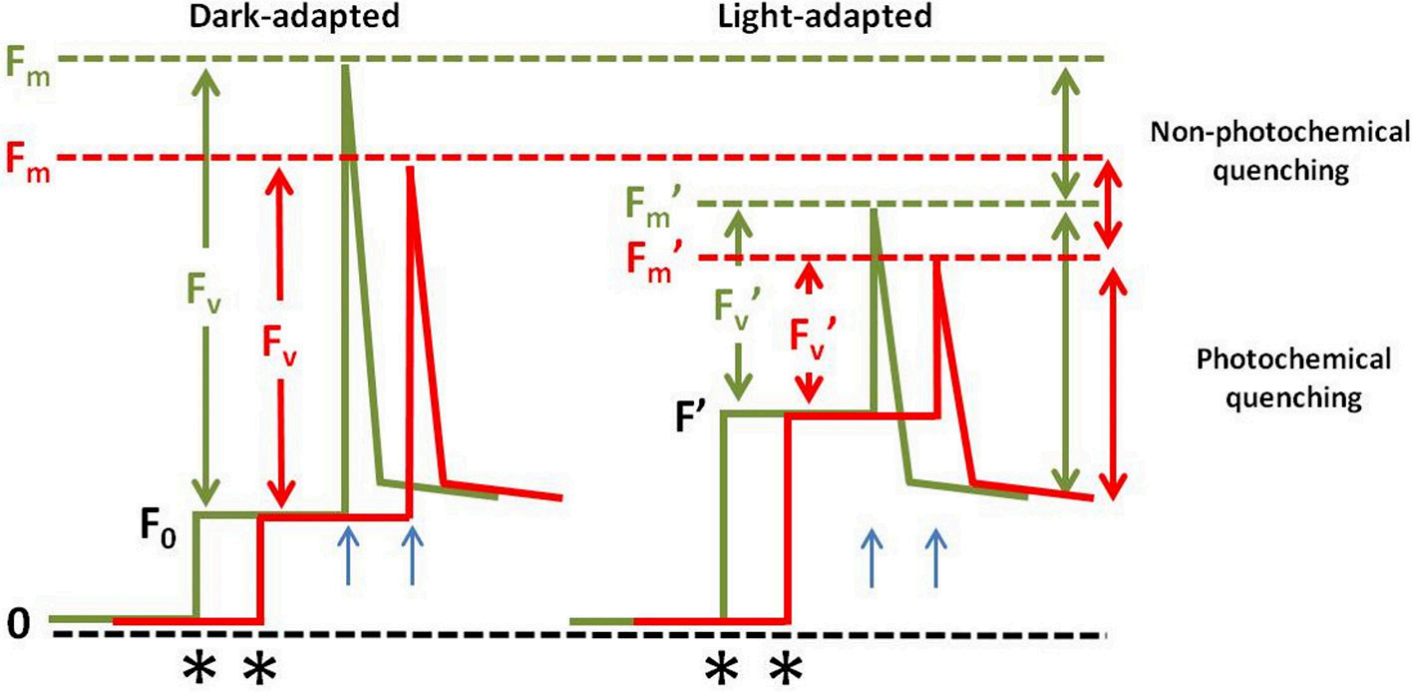
Fluorescence emission under drought stress. Fluorescence dynamics on dark- or light-adapted leaves when cultivated under normal conditions (green lines) or under drought stress (red lines). When leaves are dark-adapted the Q_A_ (Plastoquinone) is maximally oxidized and the PSII is called “open.” The exposure of the leaf to a weak measuring light (asterisks mark the point where the measuring light was turned on) results in a minimal level of measured fluorescence (F_0_). A saturating pulse is emitted (blue arrows) and F_m_, or maximum fluorescence is recorded. The difference between F_m_ and F_0_ in called F_v_ or variable fluorescence. The Fv/Fm is called maximum quantum yield of Q_A_ reduction or PSII photochemistry. When the leaf is light adapted, the minimal level of fluorescence shifts above the original background (F′). In this situation less Q_A_ is oxidized and when a light pulse is emitted the maximum fluorescence for light adapted leaves (F′_m_) is recorded and its level is lower that the F_m_ because when the plants are subjected to stress the photochemical quenching is diminished due to the photoinativation of the PSII leading to a higher level of the non-photochemical quenching (NPQ) or the dissipation of energy through heat. F′_v_ is calculated as F′_m_-F′. F′_v_/F′_m_ is called maximum PSII efficiency. This parameter is used to measure the contribution of the NPQ on the observed changes on the PSII operation.

Leaf fluorescence has been used as an indirect measure of leaf photochemical capacity. Light energy, when absorbed by the photosystem II (PSII), can be converted into photochemical energy (in the form of ATP and NAPH) or be dissipated as heat or fluorescence. Thus, all the photochemical reactions affect fluorescence emission. For example, impaired electron transport results in higher fluorescence emission (Guo and Tan, 2015). Several studies have reported diminished F_v_/F_m_ (Figure 2) when sugarcane plants experienced water stress (Silva et al., 2007, 2013; Silva M. D. A. et al., 2014; Da Graça et al., 2010). Thus, Fv/Fm ratio is an indicator of conversion of light energy into chemical energy or photochemical quenching (Silva M. D. A. et al., 2014). The reduction of photosystem II quantum yield (PSII yield or F′_v_/F′_m_) (Figure 2) has also been reported as a water stress response in sugarcane (Cha-Um and Kirdmanee, 2008; Ribeiro et al., 2013). Higher F′_v_/F′_m_ can be an indicator of a genotype with better performance under mild water deficit (Silva et al., 2007).

Root characteristics are also helpful to predict the ability of plants to adapt to drought stress (Songsri et al., 2008; Wang et al., 2009). In sugarcane, the development of deep and large root as systems can be used selection criteria for water stress tolerance (Smith et al., 2005). Higher root length density results in better water uptake, a desirable trait to extract deep soil moisture when water is limiting (Tardieu et al., 1992; Blum, 2005; Tardieu, 2012). Endres et al. (2010) found a water stress tolerant genotype with higher root length density and better field performance under water stress. Some of the potentially useful traits for improving crop productivity under water stress are listed in Tables 1, 2.

It is well established that stress signal perception and transduction and the resulting gene expression underpin all morphological, physiological and biochemical responses of plants to abiotic stresses, including water stress. Therefore, a better understanding of these cascades of molecular, cellular, tissue, organ, and whole plant responses and their interaction will help develop molecular strategies to improve plant and crop performance under water deficit.

**Figure 3:**
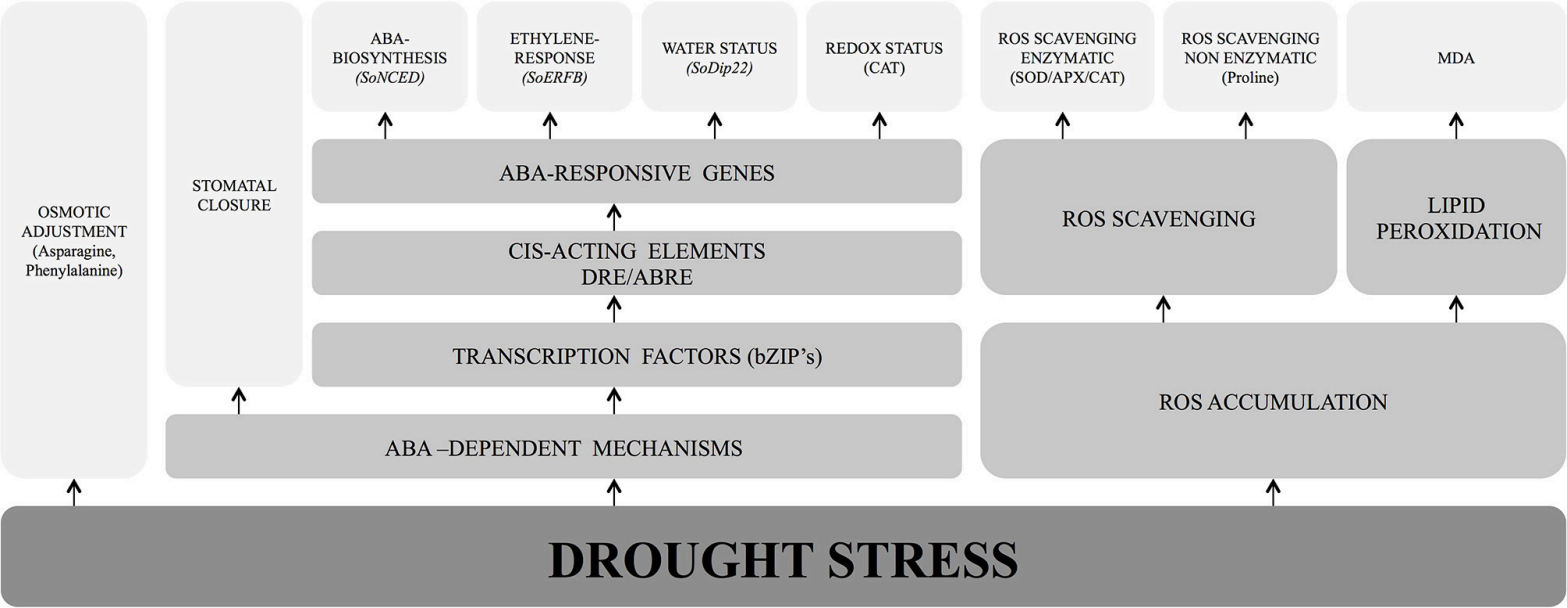
Key components of sugarcane responses to water deficit.

Unlike other major crops like rice, maize and wheat, perception and transduction of stress signaling and molecular responses in water stressed sugarcane is poorly understood. The following section summarizes the important developments in stress-related hormone signaling, response of highly damaging reactive oxygen species (ROS), enzymatic and non-enzymatic ROS scavenging, changes in amino acids profile and lipid peroxidation in sugarcane and other crops (Figure 3).

## Drought stress and ABA signaling

Abscisic acid (ABA) plays a key mediator of environmental stress signal perception and cellular stress responses in plants (Himmelbach et al., 2003). ABA is mainly biosynthesized and metabolized in vascular tissues but acts in distant cells, such as the guard cells (Umezawa et al., 2010) and its signaling occurs through different tissues and cell types by efflux and influx through specific transporters (Wilkinson and Davies, 2010). The accumulation of ABA under drought conditions triggers multiple adaptive responses. For instance, ABA has been implicated to stomatal closure, reduction of leaf and stem growth, production of deeper root system, higher root and shoot hydraulic conductivity, assimilate remobilization, induction of senescence, maintenance of turgor pressure, expression of antioxidant proteins and seed dormancy (Zeevaart and Creelman, 1988; Chaves et al., 2003; Parent et al., 2009; Han and Yang, 2015).

In relation to stomatal physiology, ABA is one of the most important regulatory signaling molecules (Dodd, 2003; Tanaka et al., 2005; An et al., 2016). Li et al. (2016) reported a gradual decline in stomatal conductance and transpiration rate with a concomitant increase in ABA content in sugarcane exposed to increasing water deficit imposed over a period of 9 days. It is known that the stomatal closure in plants can be induced by endogenous and exogenous ABA supply (Wilkinson and Davies, 2002) via one of several signaling pathways (Neill et al., 2008), involving other intermediate molecules, including secondary metabolites and ions (Li et al., 2006; Wang and Song, 2008; An et al., 2016). For example, an increase in H_2_O_2_ mediated by ABA signaling pathway raises cytosolic Ca^2+^ concentration in guard cells, inducing stomatal closure (Pei et al., 2000).

Redox changes mediated by ABA also interfere with physiological antioxidant defense system (Zhang A. et al., 2006). Jiang and Zhang (2002) demonstrated that the inhibition of ABA biosynthesis reduces ROS generation and the expression of antioxidant enzymes in maize leaves under water stress. In addition, several authors have suggested that H_2_O_2_ has an important intermediary role in ABA signal transduction and the induction of antioxidant gene expression (Guan et al., 2000; Jiang and Zhang, 2001, 2002), since it promotes H_2_O_2_ production, which in turn activates the mitogen-activated protein kinases (MAPK) signaling pathway (Zhang A. et al., 2006).

ABA-mediated responses to environmental stresses involve *cis*-acting elements and *trans*-acting factors (Shinozaki and Yamaguchi-Shinozaki, 2007). ABRE (ABA responsive element) works as a *cis*-acting DNA element in ABA-regulated gene expression and is related to dehydration responses (Shinozaki and Yamaguchi-Shinozaki, 1997; Narusaka et al., 2003). The DRE (dehydration-responsive element) acts together with ABRE in response to ABA to induce the *rd29A* gene (stress-responsive gene) (Narusaka et al., 2003). The *rd29A* promoter from *Arabidopsis* is active in sugarcane (Wu et al., 2008), suggesting the pathway is conserved between the two species. Basic leucine zipper (bZIP) transcription factors regulate physiological and stress responses. According to Schlögl et al. (2008), ABA regulates six sugarcane bZIPs, with two genes being up-regulated (*ScbZIP29* and *ScbZIP31*) and four genes down-regulated (*ScbZIP21, ScbZIP24, ScbZIP70*, and *ScbZIP79*) in sugarcane plants exposed to ABA *in vitro*. The authors suggested a model where ABA-activated kinase proteins activate or stabilize bZIPs proteins by phosphorylation, which in turn could bind to *cis*-elements (e.g., ABRE and/or DRE), controlling gene expression (Schlögl et al., 2008).

Among ABA-responsive genes involved in drought response in sugarcane, *SoNCED* encodes a 9-cis-epoxycarotenoid dioxygenase that controls a rate-limiting step in ABA biosynthesis and is induced in leaves and root tissues during stress enhancing ABA accumulation (Li C.-N. et al., 2013). *SoDip22* (sucrose-phosphate synthase) is involved in the regulation of water status in bundle sheath cells (Sugiharto et al., 1997). The *ScCAT1* (catalase) gene protects against ROS induced by environmental stimuli (Su et al., 2014). The *SodERF3* gene, encoding a transcriptional regulator of ethylene response, when overexpressed in tobacco resulted in taller plants (Trujillo et al., 2008).

These data indicate that sugarcane shares the mechanisms controlled by ABA that allow plants to adapt to and tolerate water stress. This knowledge can be used for crop improvement and development of genotypes with superior performance under water deficit conditions.

## ROS accumulation and antioxidant activity

Reactive oxygen species (ROS) are byproducts of metabolic reactions in plants, such as photosynthesis and respiration (Ahmad et al., 2010). Their biological function depends on the amount of molecules produced and their removal by cellular scavenging mechanisms (Mittler et al., 2011). ROS are key regulators of several processes, including growth, development, response to biotic and abiotic stimuli and programmed cell death (Mittler et al., 2011; Baxter et al., 2014; Luis, 2015). When plants are subjected to long periods of drought stress, changes in cellular homeostasis induce ROS outburst. When the ROS production overrides antioxidant mechanisms, it causes damage to cell membranes, DNA and proteins, which may result in cell death (Cruz de Carvalho, 2008; Gill and Tuteja, 2010; Miller et al., 2010). However, at low levels, ROS are involved in relaying stress signal to activate acclimation and defense pathways (Mittler et al., 2004).

The antioxidant defense system in plants is divided into enzymatic and non-enzymatic ROS scavenging. The superoxide dismutase (SOD) activity plays the first line of enzymatic ROS scavenging, catalyzing the conversion of ${\text{O}}_{2}^{-}$ into H_2_O_2_ within the cell. In sugarcane, SOD activity is genotype-dependent and is also modulated by water stress (Hemaprabha et al., 2004; Jain et al., 2015; Sales et al., 2015; dos Santos et al., 2015; dos Santos and de Almeida Silva, 2015). Moreover, (Jangpromma et al., 2012) showed that sugarcane cultivars classified as drought-tolerant exhibit higher levels of SOD activity under water deficit conditions. Considering that sugarcane cultivars display different SOD isoforms with complex expression patterns, it is speculated that some SOD isoforms may display a major effect on antioxidant response in sugarcane plants during stress conditions (Cia et al., 2012; Boaretto et al., 2014).

The second point of antioxidant enzymatic defense includes the hydrogen peroxide scavenging (Mittler, 2002; Cruz de Carvalho, 2008). Ascorbate peroxidase (APX) and catalase (CAT) activities regulate redox levels in cells (Luna et al., 2005) and may contribute to the increased capacity of some sugarcane cultivars to decompose H_2_O_2_ under drought conditions (dos Santos and de Almeida Silva, 2015). APX activity is increased in sugarcane cultivars evaluated under water deficit, while the relationship of CAT activity and drought tolerance is not clear (Cia et al., 2012; Boaretto et al., 2014; dos Santos and de Almeida Silva, 2015; dos Santos et al., 2015; Sales et al., 2015).

Non-enzymatic antioxidant molecules can work synergistically with enzymatic ROS scavenging mechanisms to protect plant cells against oxidative damage. The non-enzymatic system is composed by ascorbic acid (AA), reduced glutathione (GSH), α-tocopherol, carotenoids, phenolics, flavonoids and proline (Gill and Tuteja, 2010; Das and Roychoudhury, 2014). Proline (Pro) is an efficient scavenger of OH^.^ and ^1^O_2_. Furthermore, Pro can function as a compatible osmolyte, molecular chaperone and carbon and nitrogen reserve and balances cytosolic pH (Verbruggen and Hermans, 2008; Das and Roychoudhury, 2014). During water stress, Pro is accumulated in plants mainly due to increased synthesis and reduced degradation. Pro biosynthesis from glutamate is catalyzed by the enzymes Δ^1^-pyrroline-5-carboxylate (P5C) synthetase (P5CS) and P5C reductase (P5CR). Alternatively, Pro can be formed from ornithine that is converted into P5C/GSA *via* ornithine-δ-aminotransferase (OAT) (Liang et al., 2013; Bhaskara et al., 2015).

According to Guimarães et al. (2008) free-proline content is correlated to water stress tolerance in different sugarcane cultivars. Transgenic sugarcane plants expressing a heterologous *P5CS*, when grown under water deficit showed a positive correlation between enhanced proline content, increased biomass yield and photoquemical efficiency of photosystem II (Molinari et al., 2007). The authors also suggest that stress-inducible Pro accumulation in transgenic plants acts as a component of antioxidant defense system rather than an osmoregulator. In contrast, Iskandar et al. (2011) showed that Pro has the lowest increased level among other amino acids in mature sugarcane culms under water deficit. According to these authors, concentration of all amino acids was increased after water stress, especially asparagine, in young and mature sugarcane culms. The Pro concentration supports its role as an antioxidant, while other amino acids act as osmoprotectant during sucrose accumulation in the culms. The accumulation of free amino acids is commonly found in plants under stresses (Patade et al., 2008; Pagariya et al., 2012), raising the osmotic pressure, and therefore, operating as an osmoregulator (Venekamp et al., 1987; Molinari et al., 2004; Boaretto et al., 2014).

Taken all together, a correlation between water stress and the response of the antioxidant system in sugarcane is evident. The activity of ROS-scavenging enzymes may be used as a marker of water stress tolerance in sugarcane.

## Lipid peroxidation

The peroxidation of lipids is the most obvious symptom of oxidative stress in plants, caused by ROS (Huang et al., 2012). The most common lipid peroxidation pathway includes O_2_ molecules originated from photosystem II (PSII). These molecules are incorporated into plastid membranes and catalyzed by lipoxygenases (LOX) into LOOH (lipid hydroperoxide), which makes the membrane vulnerable to fragmentation and leads to a cascade of damaging events (Barclay and McKersie, 1994; Skorzynska-Polit, 2007). The fragmentation process can trigger and propagate new radicals. For instance, peroxyl radicals (LOO^*^) generates a high number of intermediates during lipid peroxidation, changing membrane structure and causing severe membrane damage or cell death (Scandalios, 1993; Spiteller, 2003). Malondialdehyde (MDA) is one of different products of this process and causes changes in cell membrane properties, such as fluidity, ion transport and enzyme activity (Sharma et al., 2012). Thiobarbituric acid reactive substances (TBARS) are extensively used to monitoring changes in the level of aldehydic products (MDA and 4-HNE) and is accepted as a marker of oxidative stress in plants (Hodges et al., 1999; Shulaev and Oliver, 2006; Pagariya et al., 2012).

High levels of H_2_O_2_ accompanied by an increase in lipid peroxidation were observed in young sugarcane plants during the initial growth phase under severe water stress (Boaretto et al., 2014). In addition, a correlation between water stress tolerance and lower levels of lipid peroxidation was reported (Cia et al., 2012; Sales et al., 2015). According to Abbas et al. (2014) lipid peroxidation may be a good parameter to identify water stress tolerant sugarcane varieties.

## Differential gene expression under drought stress

In order to better understand the molecular basis of the physiological responses of sugarcane under stress conditions, high throughput gene expression studies have been conducted (Papini-Terzi et al., 2005, 2009; Rocha et al., 2007; Rodrigues et al., 2011). Such studies have focused on signal transduction and the role of phytohormones, since water stress elicits extensive signal transduction network, involving various transcription factors, protein kinases and phosphatases (Zhu, 2001, 2002; Singh et al., 2002; Xiong et al., 2002; Rabbani et al., 2003). Additionally, as part of the plant responses to water deficit is ABA-dependent, both water stress and exogenous ABA treatment share several differentially expressed genes (Seki et al., 2002; Himmelbach et al., 2003; Shinozaki and Yamaguchi-Shinozaki, 2007).

Due to its complex genome (highly polyploidy and aneuploidy with an estimated genome size of 7,440 Mb), ESTs collections are an important tool to identify sugarcane genes and assess their function. The Brazilian Sugarcane Expressed Sequence Tags (ESTs) Sequencing Project (SUCEST) was a breakthrough in the analysis of sugarcane genes. Back in 2003, the project sequenced over 238,000 ESTs from different sugarcane tissues and cultivars that were grouped into approximately 43,000 SAS (Sugarcane Assembled Sequences) (Vettore et al., 2003), providing the basis for important transcriptome studies (Nogueira et al., 2003; Papini-Terzi et al., 2005; Camargo, 2007; Rocha et al., 2007; Rodrigues et al., 2009, 2011). Among them, hybridization-based approaches using DNA arrays have been the most successful one for large-scale gene expression profiling in sugarcane under water stress (Rocha et al., 2007; Papini-Terzi et al., 2009; Rodrigues et al., 2009, 2011; Lembke et al., 2012).

Rocha et al. (2007), Rodrigues et al. (2011), and Li et al. (2016) used cDNA arrays to study gene expression profile in leaves of sugarcane plants subjected to different water stress conditions (Table 3). Despite the differing experimental conditions several classes of genes involved in cellular metabolism (e.g., cell wall, amino acid, lipid and protein metabolism), signal transduction (transcription factors, hormone signaling proteins, calmodulins, and kinases), transport (ABC transporter, lipid transfer protein, aquaporins), hormone biosynthesis (proteins involved in the syntheses of auxin and ABA), stress responses (heat shock proteins, peroxidase) showed substantial similarity in their expression. However, the expression pattern of many genes showed considerable differences, possibly reflecting the intensity of the water deficit experienced by the test plants.

**Table 3:** Experimental conditions of large-scale gene expression assays in sugarcane under drought.

**Parameters**	**Rocha et al. (2007)**	**Rodrigues et al. (2011)**	**Li et al. (2016)**	**Rodrigues et al. (****2009****)**	
Genotype	SP90-1638	SP83-2847	GT21	SP83-5073	SP90-1638
Age (DAP)	90	60	150	60	60
Experimental conditions	G	G	G	G	G
Stress applied	Water withholding	Water withholding	Water withholding	Water withholding	Water withholding
Time of stress (hours)	24, 72, and 120	72, 192, and 240	72, 168, and 216	72, 192, and 240	72, 192, and 240
Hybridization-based approach	1,545 SAS (microarray)	3,575 ESTs (macroarray)	15,593 genes (microarray)	3,575 ESTs (macroarray)	3,575 ESTs (macroarray)
Differentially expressed sequences	93 genes	1,670 ESTs	1,501 genes	165 genes	432 genes

*DAP, days after planting; SAS, sugarcane assembled sequence; ESTs, expression sequence tags; G, greenhouse*.

In an attempt to find a correlation between drought tolerance and gene expression Rodrigues et al. (2009) compared two genotypes, classified as water stress tolerant (SP83-5073) and sensitive (SP90-1638), using a macroarray containing ESTs from leaf libraries generated by the SUCEST project (Table 3). Both genotypes present an increase in the number of differentially expressed genes along the time and intensity of stress. During the experiment, the sensitive plants showed earlier symptoms of stress, such as leaf rolling and stomatal closure, while the tolerant started showing the same symptoms after moderate stress. The authors suggested that these morpho-physiological data corroborated with the gene expression profile, since the sensitive plants activate metabolic changes earlier than the tolerant plants: most of the differentially expressed genes (93.3%) were up-regulated under severe stress in the tolerant cultivar. Whereas, in the sensitive plants, 36% of the differentially expressed genes were repressed, including stress-responsive genes (e.g., heat shock proteins) and genes involved in photosynthesis. An interesting finding coming from these studies is that the largest differentially expressed functional class genes code for unknown proteins, revealing the complexity of sugarcane genome response to water deficit. This opens up a new research field to unravel the hitherto unknown genetic mechanism(s) underpinning water stress tolerance in sugarcane.

Another fact that deserves attention is the expression of antisense transcripts in sugarcane plants subjected to water stress (Lembke et al., 2012), indicating an additional layer of complexity in gene regulation under stress condition (Lapidot and Pilpel, 2006). In this context, post-transcriptional gene regulation during water stress has been described in sugarcane. For instance, analyses focused on the expression of sugarcane miRNAs under water stress conditions (Ferreira et al., 2012; Gentile et al., 2013; Thiebaut et al., 2014) demonstrate that the microtranscriptome (miRNA transcriptome) is modulated in different genotypes and growth phases to cope with different intensities of stress (Gentile et al., 2015).

It is important to highlight that most of the molecular studies were performed using pot experiments in semi-controlled conditions, withholding water for a short duration (Table 3) to mimic a severe drought scenario. According to Skirycz et al. (2011), mild conditions would favor plants maintaining growth, photosynthesis and metabolism under stress, opening a new paradigm for the identification of tolerance alleles. Such mild to moderate water deficit conditions are more realistic from a commercial crop production perspective and must be the experimental basis for new studies. To our knowledge, there is only one study that identified miRNAs associated with drought responses in field-grown sugarcane plants (Gentile et al., 2013). When the expression profiles of these field-grown plants were compared with glasshouse plants (Ferreira et al., 2012), large differences were observed. Therefore, studies with plants under field conditions certainly will provide a different set of genes and expression profiles plants compared to those that are grown under glasshouse conditions.

The interplay between drought stress and sucrose accumulation has also been investigated. When differentially expressed genes from genotypes with different Brix (sugar) levels were compared to those subjected to water stress (Rocha et al., 2007), an extensive overlap between both datasets was observed (Papini-Terzi et al., 2009). However, Iskandar et al. (2011) found evidence that the stress caused by sucrose accumulation also triggers the expression of genes that are not induced by water deficit in sugarcane. Therefore, the complexity of both phenomena involves subsets of both common and stress-specific genes.

Interestingly, despite the sugarcane transcriptome responses to water stress vary widely according to the genetic background of the test clones and the stress applied, Iskandar et al. (2011) indicated that a strong correlation between the expression of water stress-induced genes and the expression of a sequence similar to dehydrin. The dehydrin proteins are a group of late embryogenesis abundant (LEA) proteins and have a role in the protection of cellular membranes and organelles during dehydration in sugarcane (Wahid and Close, 2007). As the stress became more severe, the expression of this gene is also induced (Rocha et al., 2007) and there is no significant difference in gene expression related to sucrose accumulation (Papini-Terzi et al., 2009; Iskandar et al., 2011). For these reasons, it can be used as a molecular marker for water stress response in sugarcane experiments (Ferreira et al., 2012; Gentile et al., 2013).

As the root is the first organ to detect water deficit in the soil and signal the stress to other cells, tissues and organs, understanding the gene activity in roots of stressed plants will provide more insights to help develop research strategies to improve crop production. Vantini et al. (2015) observed differentially expressed genes between tolerant and sensitive sugarcane varieties along time (1, 3, 5, and 10 days after withholding water) in root tissues. At the beginning of the stress (1 and 3 days), genes encoding proteins with protection function (chaperones, heat shock proteins, antioxidant enzymes and protease inhibitor proteins) were induced in the tolerant variety. Gene encoding a protein involved in ABA-response, a trehalose-phosphatase synthase (enzyme involved in the synthesis of trehalose) and serine/threonine kinase receptors also showed higher expression in the tolerant variety, revealing differences between sugarcane genotypes for water stress protection and adaptation mechanisms.

It is known that water channels proteins (aquaporins) are involved in the acclimation against abiotic stress (Alexandersson et al., 2005; Maurel et al., 2008). Aquaporins are classified into four subfamilies: PIPs (plasma membrane intrinsic proteins), TIPs (tonoplast intrinsic proteins), NIPs (26 kDa intrinsic proteins), and SIPs (small basic intrinsic proteins) (Maurel et al., 2008). da Silva et al. (2013) verified the expression profile of aquaporins in sugarcane roots under water stress. Some isoforms of aquaporins (e.g., PIP1-1, NIP3-1, and SIP1-2) were exclusively up-regulated in tolerant varieties, suggesting an involvement of stress avoidance mechanisms in sugarcane.

In brief, targeted gene expression analyses have enabled the discovery of genes that are involved in water stress response in sugarcane, but it is still difficult to directly correlate their function with tolerance levels. There are no well-characterized sugarcane genetic lines or mutants available to confirm the function of genes identified by transcriptomic analysis. Also, very little is known about the translatability of glasshouse pot study results to the field conditions. Hence further investigations using forward or reverse genetic studies are necessary for functional assessment and linking them to physiological and phenotypic responses in the field.

## Relative gene expression analyses of sugarcane under water deficit

The increased use of transcriptomic approaches has been closely associated with the use real-time quantitative PCR (qRT-PCR) as a tool for data validation (Czechowski et al., 2005; Gutierrez et al., 2008). This technique has been largely used in different sugarcane transcriptome and microtranscriptome experiments (Ferreira et al., 2012; Gentile et al., 2013; Vargas et al., 2014), transgenic plant studies (Augustine et al., 2015b; Ramiro et al., 2016), and expression profile assays under stress (da Silva et al., 2013; Thiebaut et al., 2014; Yang et al., 2014). The presence of suitable internal controls is critical for real-time reliability (Bustin, 2000, 2002). Despite its extensive use, parameters to qRT-PCR data normalization remain a source of debate (Bustin, 2000, 2002; Gutierrez et al., 2008).

Among classical reference genes (25S ribosomal RNA, *GAPDH*, β-*actin* and β-*tubulin*), Iskandar et al. (2004) concluded that *GAPDH* is the best protein-coding sugarcane gene for normalization. These authors evaluated 2 sugarcane cultivars and 3 other *Saccharum* species under water deficit and salt stress. Recently, some works have combined qRT-PCR assay and statistical tools to determine the most suitable gene to be used as reference in sugarcane (Guo et al., 2014; Ling et al., 2014; Silva R. L. D. O. et al., 2014). Silva R. L. D. O. et al. (2014) analyzed the performance of 6 candidate genes in two sugarcane varieties exposed to water deficit. They observed that the genes encoding GAPDH, α-tubulin and histone H1 were the most reliable genes for normalization of gene expression analyses in sugarcane roots under water deficit stress.

In a broader analysis, Ling et al. (2014) analyzed the stability of 13 candidate reference genes across a wide range of sugarcane samples, comprising five different tissues from plants exposed to abiotic stress and hormone treatment. The authors found that genes encoding GAPDH, eEF-1α and Eukaryotic initiation factor 4 α (EIF-4α) were the most stable genes for qRT-PCR normalization. Most interestingly, they also found that a combination of some genes had a better performance in different sets of samples: *CUL* (*Cullin*) and *eEF-1*α were more suitable for normalization of hormone treatment experiments, *CAC and CUL* for abiotic stress and *CAC* (*Clathrin adaptor complex*), *CUL, APRT* (*Adenine Phosphoribosyl Transferase*), and *TIPS-41* (*Tonoplastic Intrinsic Protein 41*) for tissue samples. Guo et al. (2014) found similar results in sugarcane plants exposed to water deficit and salt stress, with *GAPDH* and *eEF-1*α being considered as good reference genes for normalization. Additionally, they found that despite the low expression of given target gene, when normalized with two or more reference genes, the expression data become less variable across the samples. Thus, the most reliable genes for normalization of sugarcane under stress are *CAC*+*APRT, GAPDH*+*eEF1*, and *CAC*+*CUL*.

Finally, the association of qRT-PCR and statistical analysis is the best tool for reference genes selection. Further, reference gene validation for each set of experiment and their use in combination are key factors to ensure a better performance of relative expression analysis under water deficit conditions.

## Engineering sugarcane for water stress tolerance

Increasing tolerance to water stress in sugarcane so far was achieved through the overexpression of target genes (Table 4). This approach also allows to identify and validate gene function, for even those that are functionally redundant (Ito and Meyerowitz, 2000; Nakazawa et al., 2001; Kondou et al., 2010; Abdeeva et al., 2012).

**Table 4:** Sugarcane drought tolerance transgenic studies.

**References**	**Promoter**	**Gene choice**	**Gene function**	**Transformation method**	**Age (DAP)**	**Time of stress (days)**	**Experimental conditions**
Zhang A. et al., 2006	p*35S* enhanced	*Tsase*	Biomolecules stabilization	Agrobacterium	90	15	G/F
Kumar et al., 2014	*p35S*	*AVP1*	Osmotic regulation	Agrobacterium	21	15	G
Reis et al., 2014	*pRab17*	*DREB2A CA*	Gene regulation	Biolistic	90	6	G
Augustine et al., 2015a	*pUbi*	*PDH45*	Nucleic acids metabolism	Agrobacterium	120	10	G
Augustine et al., 2015b	*pUbi*	*PDH4; DREB2*	Nucleic acids metabolism; gene regulation	Agrobacterium/ biolistic	120	10	G
Augustine et al., 2015c	*pUbi*	*HSP70*	Cellular componentes; stabilization	Agrobacterium	120	10	G
Ramiro et al., 2016	*pUbi*	*BI-1*	PCD regulation	Biolistic	90	21	G
Raza et al., 2016	*p35S* enhanced	*AVP1*	Osmotic regulation	Biolistic	60	180^*^	G

PCD, programmed cell death; DAP, day after planting; G, greenhouse; F, field;

* *Irrigation reduced 50%*.

Despite the large economic importance there are only a few reports of transgenic research that made some notable advancements in improving water stress tolerance in sugarcane (Table 4). In all of them, the chosen gene is associated with water stress responses or known to be conferring water stress tolerance in other species (Zhang S. Z. et al., 2006; Reis et al., 2014; Augustine et al., 2015c; Ramiro et al., 2016). In this context it is important to note that a transgenic sugarcane line carrying choline dehydrogenase, claimed to be conferring water stress tolerance, probably will become the first commercially grown transgenic sugarcane in the world (B. Sugiharto, personal communication). This enzyme is involved in the synthesis of glycine betaine, which helps maintain cell water potential by osmotic adjustment.

Water stress-induced regulatory genes are potential candidates to develop plants tolerant to water deficit (Reis et al., 2014). DREB genes constitute the first family of transcription factors that were associated with gene regulation under abiotic stresses (Moran et al., 1994). The *AtDREB2A CA* (Constitutively Active) overexpression enhanced drought tolerance in sugarcane, as demonstrated by higher relative water content (RWC), photosynthetic rate, sucrose content and bud sprouting, without any negative effect on biomass accumulation (Reis et al., 2014). Also, the overexpression of *Erianthus arundinaceus DREB2* gene increased drought and salinity tolerance in sugarcane (Augustine et al., 2015c).

The pea *PDH45* gene encodes a DNA helicase 45, a motor protein from the helicase class. This protein group plays an important role in nucleic acids duplex unwinding. In another related study Augustine et al. (2015b) had demonstrated enhanced drought and salinity tolerance with *PDH45* overexpression in sugarcane plants. However, when the *AtDREB2A* gene was co-expressed with the pea *PDH45* gene the transgenic plants showed higher tolerance to salinity but became less tolerant to water deficit compared to those expressing *DREB2* gene alone.

HSP70s are molecular chaperones involved in membrane and protein stabilization and reestablishing the normal protein conformation under stress conditions. Sugarcane transgenic plants overexpressing an *E. arundinaceus HSP70* showed enhanced water stress and salinity stress tolerance, exhibiting high membrane thermostability, RWC, gas exchange parameters, chlorophyll content and photosynthetic efficiency under water deficit and better bud germination ability under salt stress (Augustine et al., 2015a).

The manipulation of genes regulating osmotic pressure under water deficit is a potentially useful approach to study drought tolerance mechanisms (Nelson, 1994; Raza et al., 2016). The *Arabidopsis* H+-PPase (AVP1) gene encodes a vacuolar membrane protein capable of increasing vacuolar solute content by H+ uptake from the cytoplasm into the vacuoles. The *AVP1* overexpression in transgenic sugarcane improved drought and salt tolerance (Kumar et al., 2014; Raza et al., 2016) with increased RWC and leaf water, osmotic and turgor potential and size, depth and biomass of roots.

Regarding sugar metabolism, transgenic sugarcane plants overexpressing a trehalose synthase gene (*TSase*) from *Grifola frondosa* improved water stress tolerance through trehalose accumulation (Zhang S. Z. et al., 2006). The *TSase*-overexpressing lines showed higher chlorophyll content and antioxidant enzymes activity, lower plasma membrane permeability and reduced malondialdehyde content.

The BAX subfamily stands out among the proteins that regulate the induction of ROS signaling (Watanabe and Lam, 2008). The overexpression of a BAX inhibitor from *A. thaliana* (*BI-1*) enhanced tolerance to water deficit in sugarcane plants by suppressing endoplasmic reticulum-stress-induced plant cell death (Ramiro et al., 2016).

The use of constitutive promoters is the most common approach in sugarcane transformation. The cauliflower mosaic virus (*CaMV*) 35S gene promoter is widely used for plant transformation (Porto et al., 2014), producing high transgene expression levels (Dutt et al., 2014). Its effects can be increased by including additional sequences, as duplicated 35S elements (Dhadi et al., 2009). Although high levels of 35S promoter-driven transgene expression are particularly common in dicotyledonous plants (Battraw and Hall, 1990; Benfey et al., 1990) that is not the case in monocotyledonous plants (Christensen et al., 1992; Weeks et al., 1993; Gupta et al., 2001; Lakshmanan et al., 2005; Park et al., 2010). Recent studies point to ubiquitin promoters as an emerging choice for constitutive expression of transgenes in sugarcane (Lakshmanan et al., 2005), mainly due to their transgene expression levels being significantly higher compared to other promoters, such as *CaMV* 35S promoter, the rice actin *Act1* promoter (McElroy et al., 1991) and the synthetic *Emu* promoter (Last et al., 1991).

Though not commonly used conditional promoters can be more advantageous (Kizis and Lumbreras, 2001; Dutt et al., 2014), since these promoters theoretically allow the control of gene expression in specific developmental stages and tissues in response to environmental stimulus. This could reduce yield losses in stress tolerant crops, since it eliminates the negative developmental effects caused by the constitutive transgene expression under non-stressed conditions (Peleg and Blumwald, 2011). Reis et al. (2014) used the stress-inducible ABA-responsive *Rab17* gene promoter to drive *AtDREB2A CA* gene expression in sugarcane plants, avoiding plant growth problems associated with constitutive transgene expression (Kasuga et al., 1999). However, the availability of useful conditional promoters for sugarcane is limited (Chakravarthi et al., 2016).

Two main strategies are widely used to produce transgenic plants in sugarcane: direct transformation via microprojectile (biolistics) (Bower and Birch, 1992) and indirect transformation mediated by *Agrobacterium tumefaciens* (Arencibia et al., 1998). Biolistics is a common method used for sugarcane transformation (Altpeter and Sandhu, 2010) due to its simplicity and applicability to a wide range of tissues and genotypes (Lakshmanan et al., 2005). However, it presents some disadvantages as low reproducibility, integration of a large number of transgene copies (Zhangsun et al., 2007). On the other hand, *Agrobacterium*-mediated transformation has the potential to produce complete and single copy transgene insertions, stable expression, heritability and lower cost (Somers and Makarevitch, 2004; Lakshmanan et al., 2005; Zhangsun et al., 2007; Singh et al., 2013). However, its disadvantages include genotype dependency (Lakshmanan et al., 2005; Shrawat and Lörz, 2006) and low efficiency (Shrawat and Lörz, 2006). Since both methods have advantages and disadvantages, the best choice for sugarcane transformation depends on the protocol established in a laboratory, the expertise of the group and the sugarcane genotype.

Although the advancements in transgenesis in sugarcane is impressive there are still many challenges to overcome to produce commercially useful water stress tolerant transgenic sugarcane suitable for geographically diverse crop production conditions. Rapid expansion of molecular biology tools and knowledge-base, especially the outputs of sugarcane genome sequencing, and advancements in in-field high-throughput phenomics will accelerate the development of practically useful biotechnological solutions for improving sugarcane crop productivity in water-limited environments.

## A new perspective for sugarcane improvement: genome editing

Biotechnology has entered a new era where random mutagenesis will be replaced by specific and precise genome editing approaches (Griggs et al., 2013). Genome editing is the most recent technique and it is based on the activity of sequence-specific engineered nucleases and takes advantage of the DNA repair system that exists inside each cell (Kumar and Jain, 2015). These designed nucleases target specific DNA sequences and provoke double-stranded breaks (DSB) which are repaired either by non-homologous end joining (NHEJ) or homology directed repair (HDR), resulting in diverse outcomes, such as site-directed mutagenesis, gene replacement, nucleotides insertions or deletions. Zinc Finger Nucleases (ZNFs), Transcription activator-like Effector Nucleases (TALLENs) and Clustered Regularly Interspaced Short Palindromic Repeat Associated Cas9 Nuclease (CRISPR/Cas9) system are the most used tools to this end (Kumar and Jain, 2015). Among them, TALLEN has been demonstrated in sugarcane (Jung and Altpeter, 2016) and CRISPR/Cas9 has also been recently developed for targeted genome editing in sugarcane (Altpeter, personal communication).

Application of CRISPR/Cas9 in plants is very recent (Li J. F. et al., 2013; Nekrasov et al., 2013; Shan et al., 2013). This technique has been successfully used for genome editing of several plant species, such as rice (Chen et al., 2017; Li et al., 2017; Ren et al., 2017), maize (Svitashev et al., 2016; Shi et al., 2017; Zong et al., 2017) and even in the hexaploid wheat (Gil-Humanes et al., 2017). Multiplex genome editing has also been demonstrated successfully from two genes belonging to the same family in Arabidopsis (Li J. F. et al., 2013) to simultaneous targeting 14 distinct genome loci with no detectable off-target events (Peterson et al., 2016).

Only one study so far has reported the use of CRISPR/Cas9 technique to target a gene related to drought stress, the maize gene *AGOS8*, a negative regulator of ethylene response (Shi et al., 2017). In an elegant strategy, the native *AGOS8* promoter was replaced by the *GOS2* promoter, also from maize, that confers a constitutive expression in several tissues. Mutant plants had higher levels of AGOS8 transcripts and increased grain yield under water defict and no yield penalty under well-watered conditions.

The use of CRISPR/Cas9 in crop plants is particularly interesting due to the regulatory issues involving the release of commercial products from genetically modified organisms (GMO). Recently, USDA-APHIS confirmed that a mushroom with an edited version of a gene encoding a polyphenol oxidase (to avoid browning) will not be regulated as a GMO (Waltz, 2016). This will result in a tremendous reduction of regulatory costs for cultivar development. Regulatory authorities in other countries including Brazil are still discussing regulatory frameworks for genome edited plants. In the meantime, delivery methods have been developed that introduce targeted mutations without any transgenic footprint of the genome editing tool (Liang et al., 2017).

In summary, the data so far on CRISPR/Cas9 in plants suggest that genome editing in a complex polyploidy as sugarcane may be feasible. This transformative technology will allow a paradigm shift in crop improvement and streamline regulatory approval of genetically modified sugarcane.

## Conclusion

Sugarcane is one of the first crops successfully transformed and numerous transgenes have been expressed in diverse genetic backgrounds. Yet commercial transgenic crop development is lagging behind compared to other broad-acre crops like soybean, cotton and canola. Nonetheless, it is important to note that water stress tolerance probably will become the first transgenic trait commercialized in sugarcane. Sugarcane being a vegetatively propagated crop is an ideal candidate for transgenic improvement. It can be transformed by different methods of transformation with biolistics being the most popular, and it is routinely used in many laboratories.

A large number of commercial Agribiotech companies heavily invested in developing commercial transgenic sugarcane, targeting herbicide tolerance, insect pest management, drought tolerance, biomass conversion and sucrose accumulation as priority traits. However, regulatory and marker hurdles are discouraging continued investment and commercialization of transgenic sugarcane globally. Nonetheless, there are large research programs on developing water stress tolerant sugarcane through various strategies including transgenic research are underway in Brazil, India, China, Thailand and other sugarcane producing countries. The technological innovations in molecular biology and biotechnology, the pace of gene discovery and expanding knowledge about plant and crop's response to water stress response and climate change are expected to accelerate this area of research. The discovery of stress-induced transcription factors and promoters, and the demonstration of their effectiveness in moderating the negative impact of water stress on plant growth are very encouraging and these findings in model species or other crops are now being tested in sugarcane. The research programs in Brazil, India and China using DREB transcription factors are well advanced and showing very encouraging results at controlled conditions and field level. It appears that establishing effective, useful screening methodology for transgenic plants, taking into account the real world commercial production conditions, remains a significant bottleneck in assessing water stress tolerance in sugarcane. It is anticipated that improved germplasm by a combination of transgenic and conventional breeding, matched by appropriate crop management practices will make a significant impact in productivity improvement and yield stability for commercial crop production.

## Author contributions

Contributed equally to this work: TF, MT, and DB. Contributions to the conception or design of the work: TF, MT, DB, LM, PA, GG, GR, and VG. Drafting the work: TF, MT, DB, LM, PA, GG, GR, and VG. Manuscript writing and critical review: MM, TF, MT, DB, and PL. Final approval of the version to be published: MM. Agreement to be accountable for all aspects of the work in ensuring that questions related to the accuracy or integrity of any part of the work are appropriately investigated and resolved: MM, TF, MT, DB, LM, PA, GG, GR, VG, and PL.

### Conflict of interest statement

The authors declare that the research was conducted in the absence of any commercial or financial relationships that could be construed as a potential conflict of interest.
